# Human Surfactant Protein D Suppresses Epithelial-to-Mesenchymal Transition in Pancreatic Cancer Cells by Downregulating TGF-β

**DOI:** 10.3389/fimmu.2018.01844

**Published:** 2018-08-15

**Authors:** Anuvinder Kaur, Muhammad Suleman Riaz, Shiv K. Singh, Uday Kishore

**Affiliations:** ^1^Biosciences Division, College of Health and Life Sciences, Brunel University London, Uxbridge, United Kingdom; ^2^Department of Gastroenterology and Gastrointestinal Oncology, University Medical Center, Gottingen, Germany

**Keywords:** surfactant protein, surfactant protein-D, pancreatic cancer, epithelial-to-mesenchymal transition, metastasis, transformation growth factor

## Abstract

Human surfactant protein-D (SP-D), an innate immune pattern recognition soluble factor, is known to modulate a range of cytokines and chemokines, such as TNF-α and TGF-β at mucosal surfaces during infection, allergy, and inflammation. A recent study has shown that treatment with a recombinant fragment of human SP-D (rfhSP-D) for 48 h induces apoptosis in pancreatic cancer cells. Our hypothesis is that at earlier time points, SP-D can also influence key cytokines as a part of its putative role in the immune surveillance against pancreatic cancer, where the inflammatory tumor microenvironment contributes to the epithelial-to-mesenchymal transition (EMT), invasion, and metastasis. Here, we provide the first evidence that rfhSP-D can suppress the invasive-mesenchymal properties of highly aggressive pancreatic cancer cells. Mechanistically, rfhSP-D inhibited TGF-β expression in a range of pancreatic cancer cell lines, Panc-1, MiaPaCa-2, and Capan-2, thereby reducing their invasive potential. Smad2/3 expression diminished in the cytoplasm of rfhSP-D-treated cells as compared to the untreated control, suggesting that an interrupted signal transduction negatively affected the transcription of key mesenchymal genes. Thus, expressions of Vimentin, Zeb1, and Snail were found to be downregulated upon rfhSP-D treatment in the pancreatic cancer cell lines. Furthermore, blocking TGF-β with neutralizing antibody showed similar downregulation of mesenchymal markers as seen with rfhSP-D treatment. This study highlights yet another novel innate immune surveillance role of SP-D where it interferes with EMT induction by attenuating TGF-β pathway in pancreatic cancer.

## Introduction

Pancreatic ductal adenocarcinoma (PDA) is one of the most lethal of all human malignancies with a dismal 5-year survival rate of below 7%; it is estimated to become the third leading cause of cancer-related death by 2030 (1). PDA is associated with a high rate of mortality due to metastasis, resistance to conventional chemotherapy, and high tumor recurrence rate after surgery (2). Metastasis requires epithelial-to-mesenchymal transition (EMT), which is considered a principal cause of tumor recurrence and resistance to conventional therapies (3–6). EMT is a complex and hierarchical process during tumor progression; it enables the tumor cells to acquire increased motility, leading to invasion of adjoining tissue and infiltration to systemic circulation and subsequent penetration into the adjacent tissues, resulting in macroscopic secondary tumors (7, 8). A number of EMT markers appear to regulate the invasion-metastasis process in the pancreatic tumor cells. Aberrant activation of EMT has been attributed to over-expression of mesenchymal markers, such as Zeb1 (zinc finger E-box binding homeobox 1) (9), Snail (10), and Vimentin (10), as well as repression of E-cadherin, an epithelial marker (6) in the pancreatic cancer cells. During the EMT process, E-cadherin expression is lost whereas mesenchymal markers, including fibronectin and Vimentin, are over-expressed (11). Markers, such as Snail, Slug, Zeb1, Zeb2, and Twist, to name a few, have been shown to suppress E-cadherin. E-cadherin gene-deficient mice do not survive past implantation (12). The Snail-expressing metastatic tumors are associated with poor prognosis, and drug and immune resistance, offering limited opportunity for therapeutic intervention (12).

Recent studies have shown that an inflammatory tumor microenvironment influences early tumor dissemination, EMT, and metastasis in pancreatic cancer (5, 6, 13). Pro-tumorigenic cytokines, including TGF-β, have also been linked to EMT, invasion, metastasis, and drug-resistance in many types of cancer (14, 15). In PDA, elevated TGF-β expression has been associated with a highly invasive (metastatic) phenotype, acquired through SMAD signaling. Importantly, TGF-β signaling regulates EMT-gene signatures, thereby promoting cell motility and invasiveness in the pancreatic cancer cells (8, 16–19).

Human surfactant protein-D (SP-D), a soluble collagen containing C-type lectin (collectin), is a potent innate immune molecule, found at the pulmonary and extra-pulmonary mucosal surfaces. While acting as a link between innate and adaptive immunity, SP-D is known for its role in immune surveillance and immunomodulation in infection and allergy (20). SP-D has been considered for quite some time as a modulator of inflammatory response. However, recent studies have shown its anti-proliferative properties against cancer cells (21, 22). In addition, SP-D deficiency in animal models has been shown to trigger serious adverse pathological consequences as in emphysema (23), chronic and infectious lung diseases (24–26), Crohn’s disease, and ulcerative colitis (27). A new dimension to the defense mechanism attributable to SP-D became evident when a recombinant fragment of human SP-D (rfhSP-D) comprising homotrimeric neck region and carbohydrate recognition domain (rfhSP-D) was found to selectively induce apoptosis in the sensitized eosinophils derived from allergic patients, whereas eosinophils derived from healthy individuals were unaffected (28). Proteomics analysis of an eosinophil-like leukemic cell line (AML14.3D10), treated with rfhSP-D, showed that it caused cell cycle arrest *via* activation of G2/M checkpoints, and subsequently induced apoptosis *via* p53 pathway (21). Treatment of human lung adenocarcinoma A549 cell line with SP-D has been shown to suppress the epidermal growth factor (EGF) signaling by interrupting the EGF–EGFR interaction, thus reducing cell proliferation, invasion, and migration (22). Recently, Kaur et al. have shown that treatment with rfhSP-D for 48 h differentially induced apoptosis in pancreatic cancer cell lines, such as Panc-1, MiaPaCa-2, and Capan-2 *via* Fas-mediated pathway, involving cleavage of caspase 8 and 3 (29).

In this study, we demonstrate, for the first time, an early anti-tumorigenic role of rfhSP-D, where it suppresses the EMT and invasive-mesenchymal phenotype in pancreatic cancer cell lines. We show that rfhSP-D inhibits the invasive functions of TGF-β/SMAD expressing pancreatic cancer cells. Mechanistically, rfhSP-D downregulates the EMT-related gene signatures (Vimentin, Zeb1, and Snail), and hence, pancreatic cancer cells invasion, mainly by attenuating TGF-β signaling pathway.

## Materials and Methods

### Cell Culture

Human pancreatic cancer cell lines, such as Panc-1 (CRL-1469), MiaPaCa-2 (CRL-1420), and Capan-2 (HTB-80), were obtained from ATCC, and used as an *in vitro* model in this study. All cell lines were cultured in DMEM-F12 media supplemented with 2 mM l-glutamine, 10% v/v fetal calf serum (FCS), and penicillin (100 units/ml)/streptomycin (100 µg/ml) (Thermo Fisher). All cell lines were grown at 37°C under 5% v/v CO_2_ until 80–90% confluency was attained.

### Expression and Purification of rfhSP-D

Expression and purification of a recombinant form of human SP-D was carried out as reported previously (28). Plasmid pUK-D1 (containing cDNA sequences for 8 Gly-X-Y repeats, neck and CRD region of human SP-D) was transformed into *Escherichia coli* BL21 (λDE3) pLysS strain (Invitrogen). A single colony was inoculated in 25 ml of Luria–Bertani (LB) medium containing ampicillin (100 µg/ml) and chloramphenicol (34 µg/ml) (Sigma-Aldrich) at 37°C on a shaker overnight. The overnight inoculum was grown in a 1 l LB medium (containing ampicillin and chloramphenicol) until the OD_600_ reached 0.6, induced with 0.4 mM isopropyl β-D-thiogalactoside (IPTG) (Sigma-Aldrich, UK) for 3 h at 37°C on an orbital shaker, and then centrifuged (5,000 × *g*, 4°C, 15 min). The bacterial cell pellet was lysed using 50 ml of lysis buffer (50 mM Tris–HCl, pH 7.5, 200 mM NaCl, 5 mM EDTA, pH 7.5) containing lysozyme (100 µg/ml; Sigma-Aldrich) and 0.1 mM phenylmethylsulfonyl fluoride (PMSF; Sigma-Aldrich, UK) at 4°C for 1 h. The bacterial cell suspension was sonicated at 60 Hz for 30 s with an interval of 2 min each (15 cycles) and centrifuged at 13,800 × *g* for 15 min at 4°C. The pellet containing insoluble rfhSP-D as inclusion bodies was suspended in 25 ml of solubilization buffer (50 mM Tris–HCl, pH 7.5, 100 mM NaCl, 5 mM EDTA, pH 7.5) containing 6 M urea at 4°C for 1 h and then centrifuged at 13,800 × *g* at 4°C for 15 min. The supernatant was serially dialyzed against solubilization buffer containing 4, 2, 1, and 0 M urea and 10 mM β-mercaptoethanol for 2 h at 4°C, followed by final dialysis in solubilization buffer containing 5 mM CaCl_2_ (Affinity buffer) for 3 h and centrifuged at 13,800 × *g*, 4°C for 15 min. The supernatant containing soluble rfhSP-D was passed through a 5 ml Maltose-Agarose column (Sigma-Aldrich). The affinity-column was washed extensively using affinity buffer containing 1 M NaCl (20 ml) before eluting the bound rfhSP-D protein with solubilization buffer containing 10 mM EDTA, pH 7.5. The concentration of the eluted protein was determined *via* OD_280_. The peak fractions were passed through Pierce™ High Capacity Endotoxin Removal Resin (Qiagen) to remove lipopolysaccharide (LPS). Endotoxin levels were determined using the QCL-1000 Limulus amebocyte lysate system (Lonza); the assay was linear over a range of 0.1–1.0 EU/ml (10 EU = 1 ng of endotoxin). The amount of endotoxin levels was found to be <4 pg/μg of the rfhSP-D protein.

### Cell Morphological Studies

Morphological alterations were examined in order to determine the optimal dose of rfhSP-D for the treatment of pancreatic cell lines. Panc-1 cells were seeded at a low density (0.1 × 10^4^) and grown overnight in DMEM-F12 containing 10% FCS in a 12-well plate (Nunc). The cells were washed twice with PBS and incubated in serum-free medium with and without rfhSP-D (5, 10, or 20 µg/ml). An area of 5–10 cells was selected for each treatment condition for analysis at 0, 6, and 24 h using phase contrast Axioscope microscope.

### Matrigel Invasion Assay

The invasion assay was performed using Corning™ BioCoat™ Matrigel™ Invasion Chamber (BD Matrigel Matrix). Inserts, pre-coated with basement membrane that was extracted from Engelbreth-Holm-Swarm mouse tumor, were reconstituted in serum-free DMEM-F12 at 37°C for 2 h. 35,000 cells, re-suspended in 500 µl serum-free DMEM-F12, were added to the top wells of the inserts with and without rfhSP-D (20 µg/ml), and 500 µl of medium containing serum was added to the bottom of the inserts in a 24-well plate and incubated at 37°C for 22 h. Next, medium containing non-evaded cells were discarded and remaining cells were scraped off from the membrane using a sterile cotton bud, ensuring cells at the bottom of the membrane remained intact for fixing. Fixation was done using 100% methanol for 2 min, followed by 2 min incubation with toluidine blue to stain the evaded cells. The membrane was cut using a sterile scalpel and mounted on the slide to count the evaded cells.

### Fluorescence Microscopy

Cells (0.5 × 10^5^) were grown on coverslips overnight at 37°C under 5% v/v CO_2_. Next day, cells were washed three times with sterile PBS before being incubated with and without rfhSP-D (5, 10, or 20 µg/ml) in a serum-free DMEM-F12 for 1 h for rfhSP-D binding to the cells, and 12 or 24 h for intracellular staining. The coverslips were washed three times with PBS in between each step. For rfhSP-D binding analysis, the coverslips were incubated for 1 h with mouse anti-human SP-D (a kind gift from Prof. U. Holmskov, Odnese, Denmark; 1:200) followed by goat anti-mouse IgG H&L (Cy5 ^®^, 1:500; Abcam) and Hoechst (1:10,000; Thermo Fisher) for immunofluorescence analysis. For the intracellular staining (Vimentin, Zeb1, E-Cadherin, SP-D, and TGF-β), the cells were fixed and permeabilized using ice-cold 100% methanol at −20°C for 10 min. This was followed by 1 h incubation with rabbit anti-human IgG antibody (Vimentin, Zeb1, and TGF-β) (1:500; Cell Signaling) and human SP-D (1:500), and another 1 h incubation with Alexa Fluor 488 (1:500; Thermo Fisher) and Hoechst (1:10,000; Thermo Fisher) for immunofluorescence analysis. For human pancreatic tissue, immunofluorescence was performed as described previously (30). Immunostaining was visualized using confocal laser-scanning microscope (Olympus). The human pancreatic tissue was obtained from the Department of Pathology, Philipps-University of Marburg, Germany following ethical considerations as stipulated by the University’s ethical guidelines.

### Quantitative RT-PCR

Pancreatic cancer cell lines were incubated with either rfhSP-D (20 µg/ml) or anti-TGF-β neutralizing antibody (Thermo Fisher) for various time points and the centrifuged cell pellets were stored at −80°C. Total RNA was extracted using GenElute Mammalian Total RNA Purification Kit (Sigma-Aldrich, UK), followed by DNase I treatment to remove any DNA impurities. The concentration and purity of total RNA were determined by measuring the absorbance at 260 nm and 260:280 nm ratio, respectively, using NanoDrop 2000/2000c (Thermo-Fisher Scientific). Total RNA (2 µg) was used to synthesize cDNA using High Capacity RNA to cDNA Kit (Applied Biosystems). The web-based Basic Local Alignment Search Tool and Primer-BLAST (http://blast.ncbi.nlm.nih.gov/Blast.cgi) were used to design the forward and reverse primer sequences (Table 1).

**Table 1:** Target genes and terminal primers used in the qPCR analysis.

Gene	Forward primer	Reverse primer
Snail	5′- GAGCTGACCTCCCTGTCAGA-3′	5′-GTTGAAGGCCTTTCGAGCCT-3′

Vimentin	5′-CTCTGGCACGTCTTGACCTT-3′	5′-TCTTGGCAGCCACACTTTCA-3′

Zeb1	5′-AAGGGCAAGAAATCCTGGGG-3′	5′-ATGACCACTGGCTTCTGGTG-3′

TGF-β	5′-GTACCTGAACCCGTGTTGCT-3′	5′-GTATCGCCAGGAATTGTTGC-3′

18S	5′-ATGGCCGTTCTTAGTTGGTG-3′	5′-CGCTGAGCCAGTCAGTGTAG-3′

Each qPCR reaction, carried out in triplicates, consisted of 5 µl Power SYBR Green MasterMix (Applied Biosystems), 75 nM of forward and reverse primers, and 500 ng cDNA, making up to a 10 µl final volume per well. Relative mRNA expression was analyzed using 7900HT Fast Real-Time PCR System (Applied Biosystems). Samples were initially incubated at 50°C (2 min) and 95°C (10 min), followed by amplification of the template for 40 cycles (each cycle for 15 s at 95°C and 1 min at 60°C). Human 18S RNA, an endogenous control, was used to normalize the gene expressions. The cycle threshold (Ct) mean value for each target gene was used to calculate the relative expression using the relative quantification (RQ) value and formula: RQ = 2^−ΔΔCt^, which was compared with the relative expression of the untreated cells.

### Western Blot

Cells (0.1 × 10^7^) were cultured in a 6-well plate (Nunc) and incubated with and without rfhSP-D (20 µg/ml) in a serum-free DMEM-F12 for various time points. The cells were then mixed with 2× treatment buffer (50 mM Tris–HCL pH 6.8, 2% v/v β-merceptoethanol, 2% v/v SDS, 0.1% w/v bromophenol blue, and 10% v/v glycerol) and sonicated for 30 s before running on a SDS-PAGE (12% w/v) for 90 min at 120 V. The SDS-PAGE separated proteins were electrophoretically transferred onto a nitrocellulose membrane using an iBlot 7-min Blotting System (Thermo Fisher), followed by blocking with 5% w/v non-fat dried milk powder (Sigma) in 100 ml PBS for 2 h on a rotatory shaker at room temperature. The membrane was washed with PBST (PBS + 0.05% Tween 20) three times, each time for 10 min. The membrane was then incubated with primary anti-human TGF-β antibody, (1:1,000; R&D systems) at 4°C overnight on a rotatory shaker, followed by secondary anti-rabbit IgG horseradish peroxidase-conjugate (1:1,000; Promega) for 1 h at room temperature. The positive bands were visualized using 3,3′-diaminobenzidine (DAB) substrate kit (Thermo Fisher).

### Statistical Analysis

Graphs were made and statistically analyzed using an unpaired one-way or two-way ANOVA tests in Graphpad Prism 6.0. Significant values were considered based on *p < 0.05, **p < 0.01, ***p < 0.001, and ****p < 0.0001 between treated and untreated samples. Error bars show the SEM, as indicated in the figure legends.

## Results

### Presence of SP-D Can be Detected in Human Pancreatic Cancer Tissues

**Figure 1:**
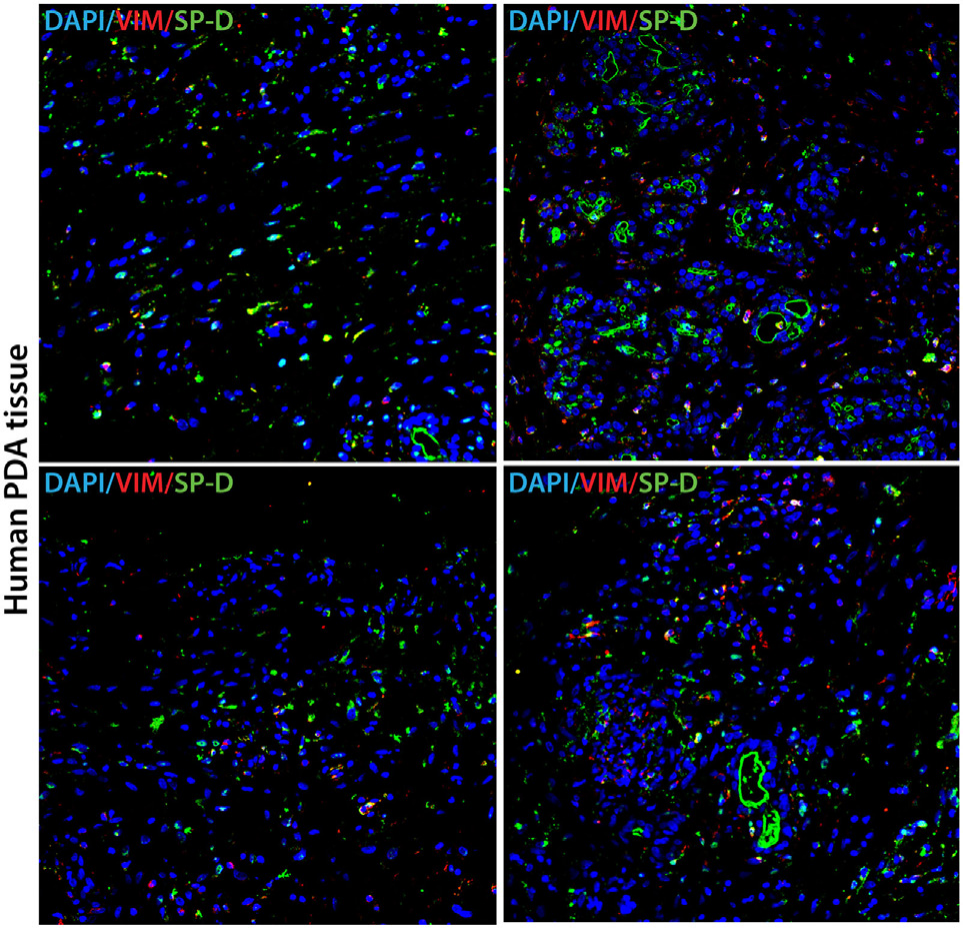
Human pancreatic cancer tissue expressing SP-D and Vimentin. Double immunofluorescence staining of SP-D (green), Vimentin (red), and DAPI (blue) in four different areas of representative human pancreatic cancer tissue. Scale bars, 50 µm.

**Figure 2:**
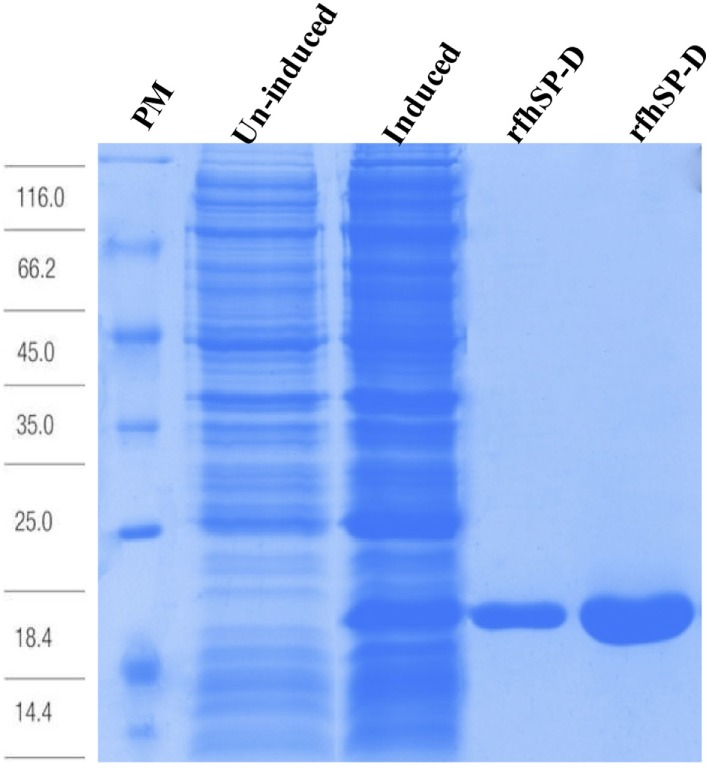
Recombinant fragment of human SP-D (rfhSP-D) expression and purification. *Escherichia coli* BL21 (λDE3) pLysS containing pUK-D1 was induced with IPTG. The expressed bacterial cells show rfhSP-D over-expression at ~20 kDa as compared to un-induced cells. The insoluble inclusion bodies were refolded and affinity purified on maltose-agarose. The peak fractions shown here appeared homogenous on a 12% SDS-PAGE.

Immunofluorescence studies of human pancreatic cancer tissues probed with anti-human SP-D monoclonal antibody showed positive staining, confirming the presence of SP-D in the tissues (Figure 1). Additionally, the tissues were probed with anti-Vimentin, which also showed positive staining. However, we could not detect or purify SP-D from the culture supernatants of all three pancreatic cancer cell lines (data not shown). It is possible that the SP-D staining pertains to the tumor microenvironment. Thus, we proceeded to test the effect of exogenous rfhSP-D (Figure 2) on cultured pancreatic cancer cell lines.

### rfhSP-D Induces Morphological Alterations in the Pancreatic Cancer Cell Line, Panc-1

**Figure 3:**
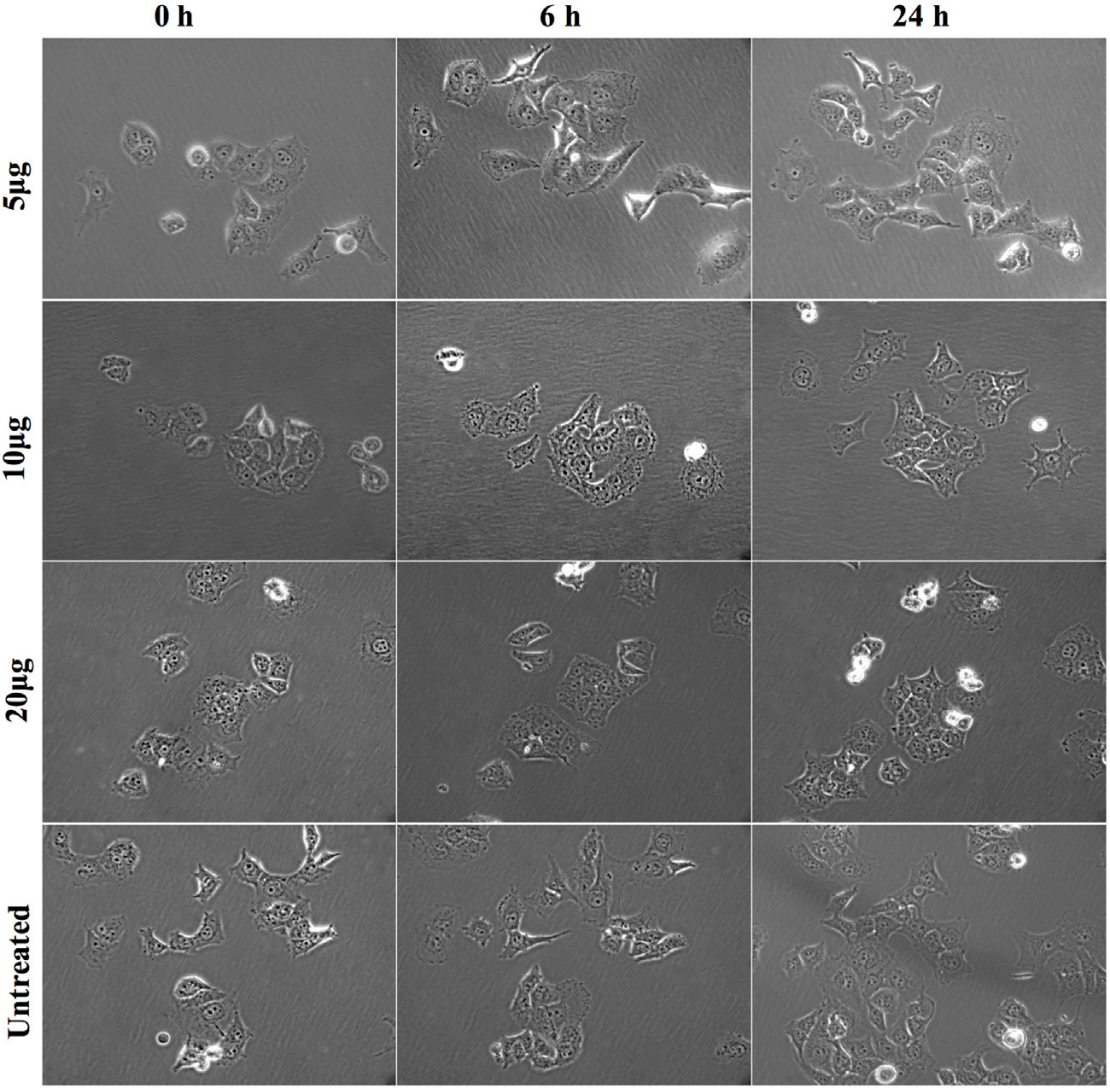
Morphological changes in Panc-1 cells following rfhSP-D treatment. Panc-1 cells (0.1 × 10^4^) were grown overnight in a 12-well plate tissue culture and colonies of 5–10 cells were selected for analysis with and without rfhSP-D (5, 10, and 20 µg/ml) at 0, 6, and 24 h. The cells treated with 5 µg/ml and untreated appeared to undergo epithelial-to-mesenchymal transition as they acquired spindle shape and showed reduced cell–cell contact, whereas cells treated with 10 and 20 µg/ml appeared static and did not acquire spindle shape.

To determine the optimal dose of rfhSP-D, Panc-1 cells were incubated with 5, 10, and 20 µg/ml for up to 24 h. The colonies of 10–15 cells were selected for each protein dose to observe the effect of rfhSP-D on cell morphology and cell division. The images of the selected cell colonies were taken at 0, 6, and 24 h (Figure 3). Untreated Panc-1 cells, as well as those treated with rfhSP-D (5 µg/ml), acquired spindle-type cell morphology, reduced cell–cell contact, and continued to divide in a time-dependent manner. However, Panc-1 cells, treated with 10 and 20 µg/ml concentration of rfhSP-D, did not acquire spindle shape and appeared to be static. However, cell morphology at 10 µg/ml appeared to be regaining the spindle shape and reduced cell–cell contact, with some evidence of cell division by 24 h. At 20 µg/ml, the non-spindle effect continued up to 24 h. Although some cell division was noted, cells remained in close contact with each other and static. Therefore, 20 µg/ml dose of rfhSP-D was selected to investigate its possible effect on EMT and invasion involving Panc-1, MiaPaCa-2, and Capan-2 cells.

### rfhSP-D Suppresses the Invasive Capacity of the Pancreatic Cancer Cell Lines

**Figure 4:**
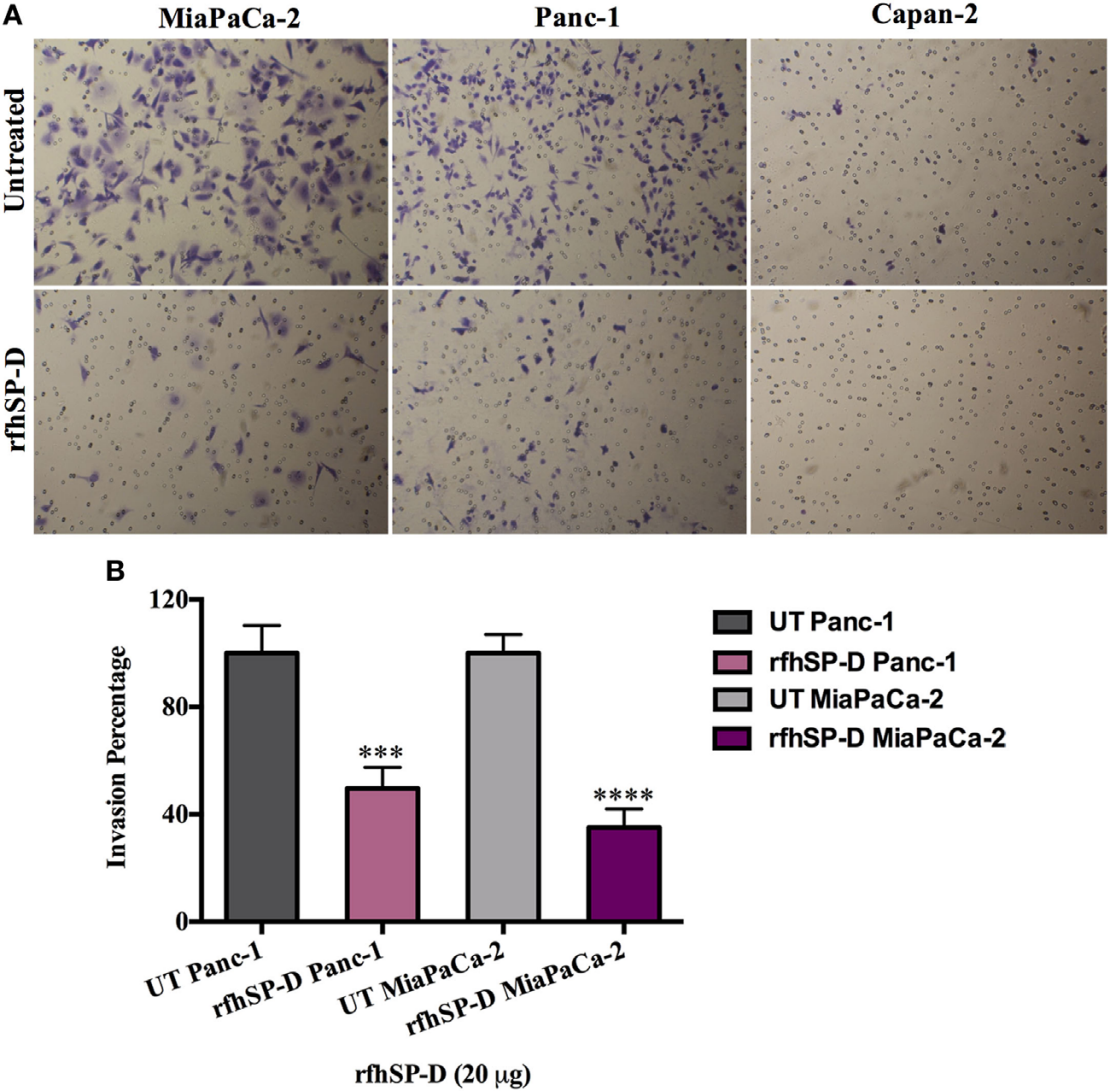
Recombinant fragment of human SP-D (rfhSP-D) supresses invasiveness in pancreatic cancer cell lines. **(A)** The cell invasion was analyzed by incubating 35,000 cells with and without rfhSP-D (20 µg/ml) in the BioCoat™ Matrigel™ Invasion Chambers at 37°C for 22 h. The invasive cells were fixed and stained before mounting the membrane on the slide for cell counting. The images show the difference between treated and untreated. **(B)** Treatment with rfhSP-D significantly reduced the cell invasion as for Panc-1 (~50%) and MiaPaCa-2 (~65%). However, as anticipated, no invasion was detected in Capan-2 cell line, neither in treated nor untreated samples. Significant values were considered based on ***p < 0.001 and ****p < 0.0001 between treated and untreated samples.

The matrigel invasive chambers, pre-coated with extracellular matrix proteins, were used to incubate the pancreatic cancer cells (3.5 × 10^4^) in the presence and absence of rfhSP-D (20 µg/ml) in the upper surface of the chamber; serum containing DMEM-F12 was used as a chemo-attractant in the bottom surface for 22 h. Both Panc-1 and MiaPaCa-2 cell lines, treated with rfhSP-D, showed significantly reduced invasion in the matrigel (Figure 4A); however, almost no invasion occurred in Capan-2 whether rfhSP-D treated or untreated. This was anticipated since Capan-2 is a low-grade cancer cell line. MiaPaCa-2 was most affected as the invasion was reduced by 65%, followed by Panc-1 cell line, which was approximately 50% less than the untreated cells (Figure 4B). Since pancreatic cancer cells are well known to overexpress TGF-β, which has a prominent-role in inducing EMT, the expression of TGF-β in the pancreatic cell lines was investigated following the treatment with rfhSP-D (20 µg/ml).

### rfhSP-D Downregulates TGF-β Gene Expression in Pancreatic Cancer Cells

**Figure 5:**
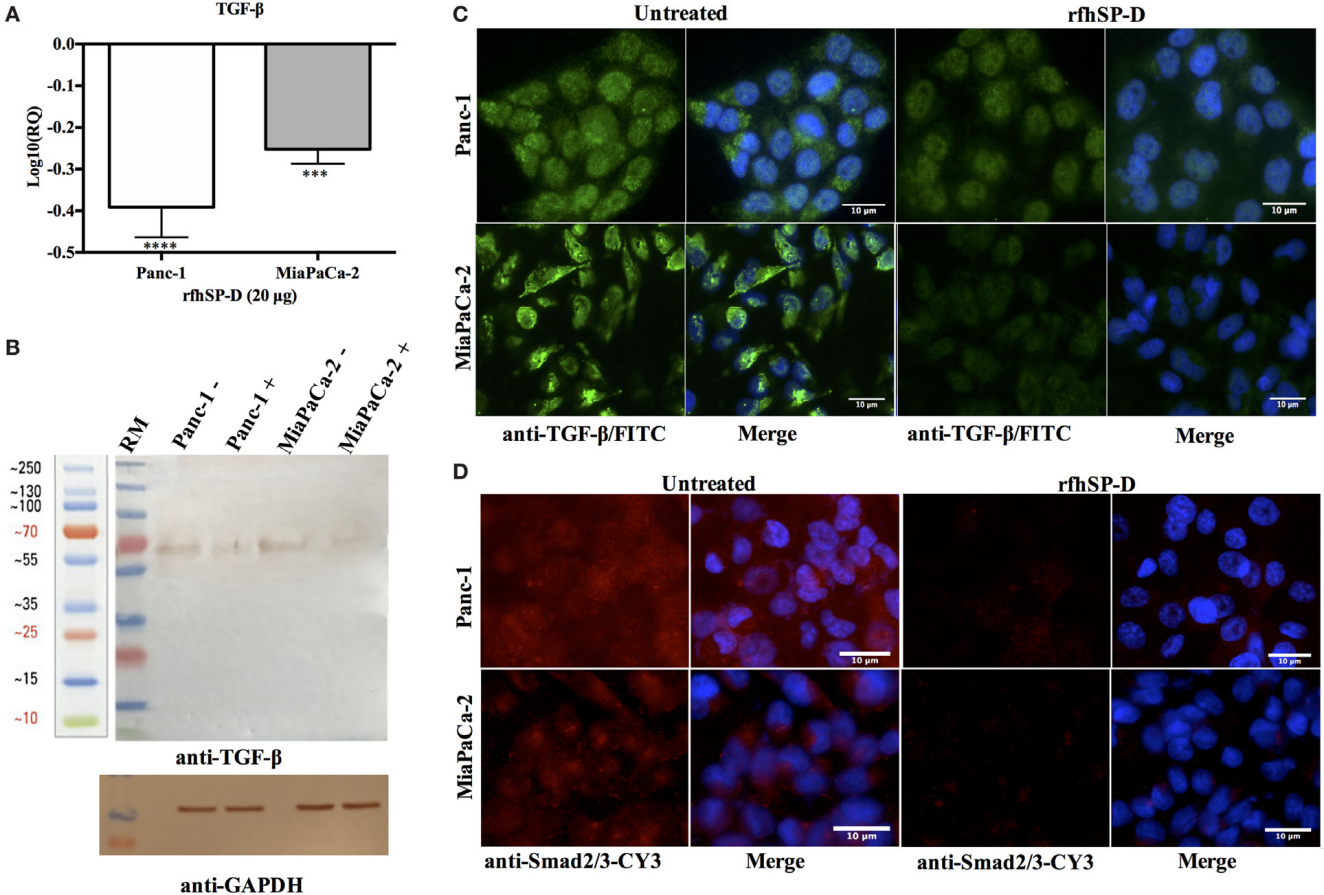
Treatment with rfhSP-D downregulates TGF-β expression. **(A)** TGF-β gene expression, as determined by qPCR, was significantly downregulated at 12 h in both Panc-1 and MiaPaCa-2 cell lines. Significant values were considered based on ***p < 0.001 and ****p < 0.0001 between treated and untreated samples. **(B)** Western blot analysis revealed a ~60 kDa band that was very faint in the rfhSP-D-treated sample. **(C)** TGF-β intracellular staining for cell lines. 0.5 × 10^5^ cells were incubated with and without rfhSP-D (20 µg/ml) for 24 h followed by probing with rabbit anti-human TGF-β1 antibody (1:500), and then by Alexa Fluor 488 (1:500) and Hoechst (1:10,000). Immunofluorescence analysis showed bright green fluorescence in the cytoplasm of the untreated cells as compared to weak staining in the treated cells. **(D)** Smad 2/3 intracellular staining for Panc-1 and MiaPaCa-2 cell lines with and without rfhSP-D (20 µg/ml) treatment for 24 h using rabbit anti-human Smad2/3 antibody (1:500) and then probed with anti-rabbit CY3 (1:1,000) showed the presence of Smad2/3 in the cytoplasm of untreated cells; however, no CY3 fluorescence was detected in the rfhSP-D-treated cells.

The transcriptional expression of TGF-β was significantly downregulated in Panc-1 (~log_10_ 0.5-fold) and MiaPaCa-2 (~log_10_ 0.3-fold) at 12 h (Figure 5A), whereas Capan-2 showed no difference following rfhSP-D treatment. This suggested that reduced TGF-β transcripts were being made following the rfhSP-D treatment. Thus, the total cell extracts for all cell lines were analyzed by Western blot using anti-human TGF-β monoclonal antibody, which revealed a reduction in the amount of TGF-β (~60 kDa band) in the rfhSP-D-treated Panc-1 and MiaPaCa-2 samples, as compared to the untreated cells; the amount of TGF-β in Capan-2 was unaffected (Figure 5B). The qualitative analysis by immunofluorescence microscopy showed that TGF-β expression at 24 h diminished considerably within the cytoplasm of Panc-1 and MiaPaCa-2 cell lines following rfhSP-D treatment (Figure 5C); however, this was not evident in the case of Capan-2 cells (result not shown). During TGF-β induced EMT pathway, Smad2/3 are phosphorylated in the cytoplasm, followed by translocation into nucleus; however, Smad2/3 staining appeared very weak in the cytoplasm of the rfhSP-D-treated Panc-1 and MiaPaCa-2 cell lines (Figure 5D). No difference was seen in Capan-2-treated and untreated cells (data not shown). Next, key regulators of EMT were examined.

### rfhSP-D Reduces the Expression of EMT Markers

**Figure 6:**
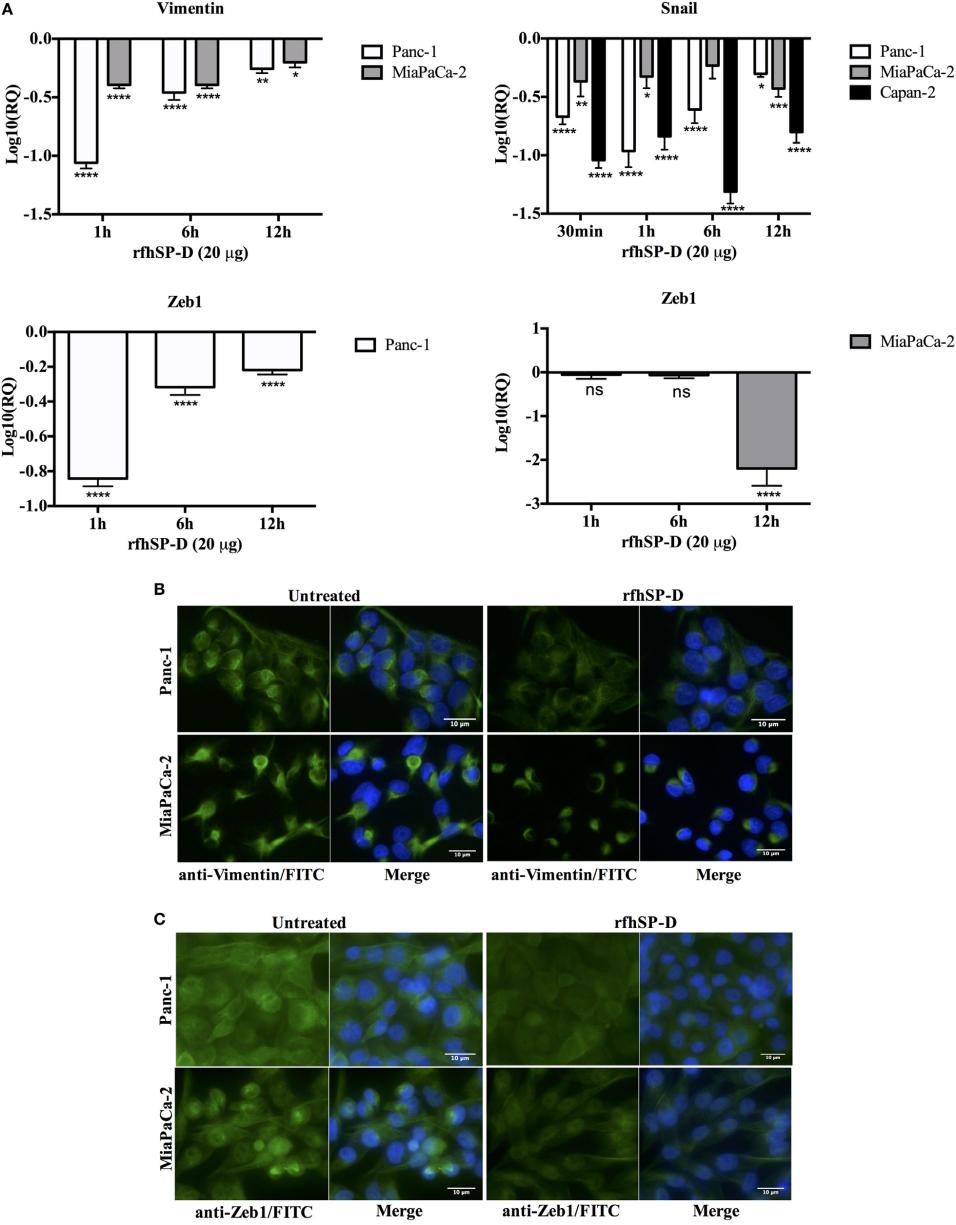

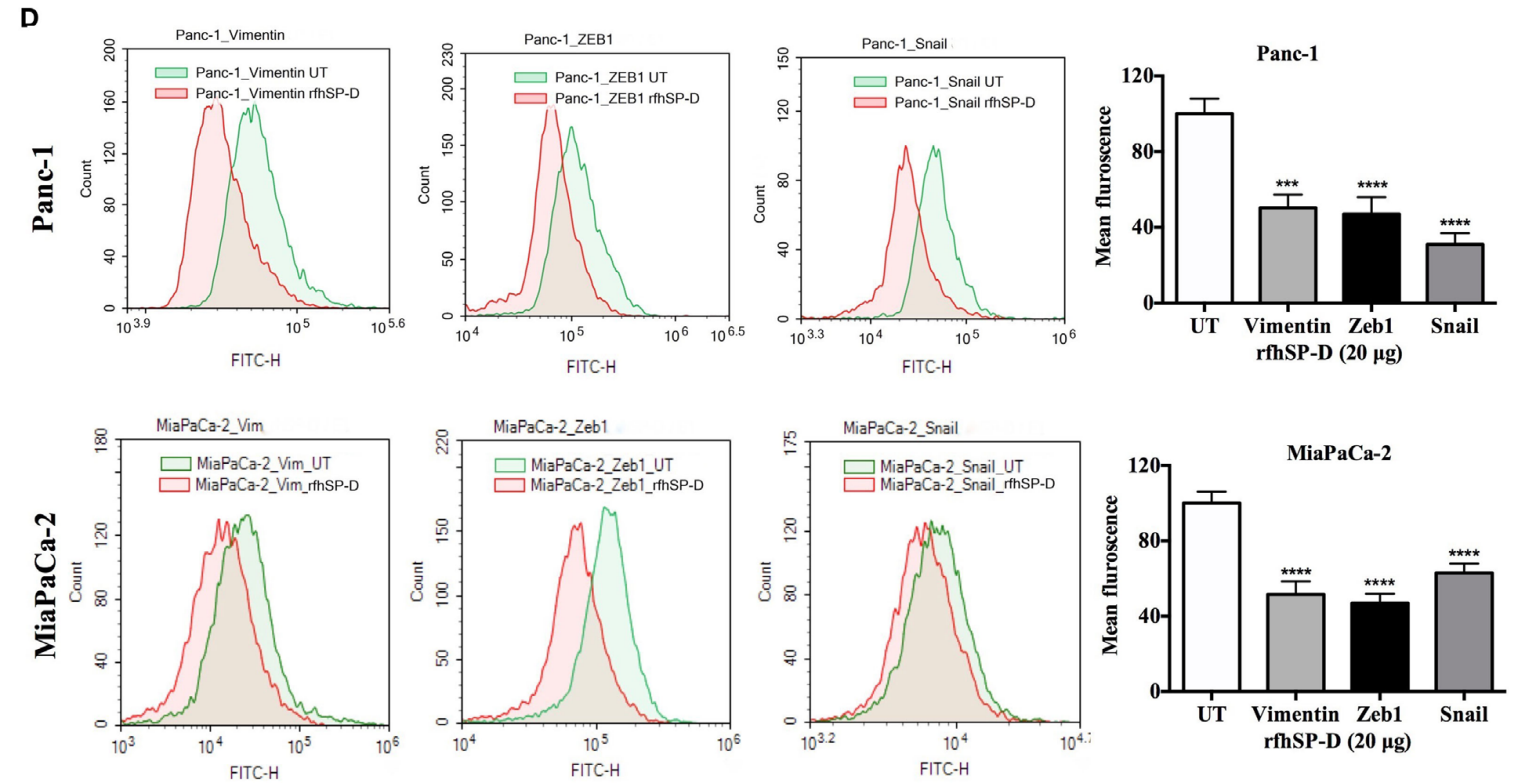
Recombinant fragment of human SP-D (rfhSP-D) downregulates the expression of epithelial-to-mesenchymal transition (EMT) markers (Vimentin, Snail, and Zeb1). **(A)** All cell lines were incubated with and without rfhSP-D (20 µg/ml) for various time points and total RNA was extracted to synthesize cDNA. Each qPCR reaction was carried out in triplicates and human 18S RNA, an endogenous control, was used to normalize the gene expressions. The cycle threshold mean was used to calculate the relative expression to compare treated and untreated. EMT inducers, Vimentin, Snail, and Zeb1, were significantly downregulated across all cell lines. Significant values were considered based on *p < 0.05, **p < 0.01, ***p < 0.001, and ****p < 0.0001 between treated and untreated samples. **(B)** Vimentin intracellular staining for all cell lines using 0.5 × 10^5^ cells incubated with and without rfhSP-D (20 µg/ml) for 24 h followed by probing with rabbit anti-human Vimentin antibody (1:500) and then with Alexa Fluor 488 conjugated with FITC (1:500) and Hoechst (1:10,000) for immunofluorescence analysis showed bright green fluorescence in the cytoplasm of the untreated cells as compared to very weak staining in the rfhSP-D-treated cells. **(C)** Zeb1 intracellular staining for all cell lines using 0.5 × 10^5^ cells incubated with and without rfhSP-D (20 µg/ml) for 24 h followed by probing with rabbit anti-human Zeb1 antibody (1:500) and then with Alexa Fluor 488 (1:500) and Hoechst (1:10,000) for immunofluorescence analysis showed bright green fluorescence in the cytoplasm of the untreated cells of all cell lines as compared to minor staining in the treated cells of Panc-1 and MiaPaCa-2. **(D)** The quantitative analysis of the mean fluorescence of Vimentin, Zeb1, and Snail of Panc-1 and MiaPaCa-2 cell lines showed a significant reduction in the rfhSP-D-treated cells as compared to untreated counterparts. Significant values were considered based on ****p < 0.0001 between treated and untreated samples.

To determine whether exogenous rfhSP-D was affecting the key regulators of EMT, we examined the gene expression of Vimentin, Zeb1, and Snail at various time points. All of these markers were differentially downregulated in all the cell lines. Vimentin was significantly downregulated in Panc-1, ~log_10_ one-fold at 1 h, and ~log_10_ 0.5-fold at 6 h (Figure 6A). MiaPaCa-2 showed ~log_10_ 0.5-fold downregulation at 1 h that remained constant at 6 h (Figure 6A). Vimentin downregulation occurred at a later time-point (6 h) in Capan-2 cell line (~log_10_ 0.5-fold) (Figure 6A). All cell lines showed a similar pattern of decrease in the transcript levels of Vimentin at 12 h compared to earlier time-points following rfhSP-D.

Snail was significantly downregulated in Panc-1 (~log_10_ onefold), MiaPaCa-2 (~log_10_ 0.5-fold), and Capan-2 (~log_10_ one-fold) at 1 h and remained downregulated at 6 and 12 h (Figure 6A). Zeb1 transcript level was significantly reduced in Panc-1 (~log_10_ 0.5-fold) at 1 and 6 h and MiaPaCa-2 (~log_10_ two-fold) at 12 h (Figure 6A). No difference in Zeb1 gene expression was seen in Capan-2. Since TGF-β regulates these EMT markers and rfhSP-D downregulates TGF-β, rfhSP-D mediated downregulation of EMT markers (Vimentin, Snail, and Zeb1) was logically evident.

Qualitative analysis of Vimentin (Figure 6B) and Zeb1 expressions (Figure 6C) in Panc-1 and MiaPaCa-2 cell lines *via* immunofluorescence microscopy revealed a significant difference in the cytoplasmic presence of these proteins in the rfhSP-D-treated cells, as compared to untreated cells. These observations were consistent with flow cytometry data that was carried out to further validate quantitatively the downregulation of the Vimentin, Zeb1, and Snail in Panc-1 and MiaPaCa-2 cells (Figure 6D) following 24 h treatment with rfhSP-D. The mean fluorescence values for Vimentin, Zeb1, and Snail were approximately 50% less in the rfhSP-D-treated cells as compared to their untreated counterparts and a clear shift was seen in the fluorescence intensity between rfhSP-D-treated and untreated cells (Figure 6D).

### Blocking TGF-β *Via* Neutralizing Antibody Reduces the Expression of EMT Markers in a Way Similar to rfhSP-D

**Figure 7:**
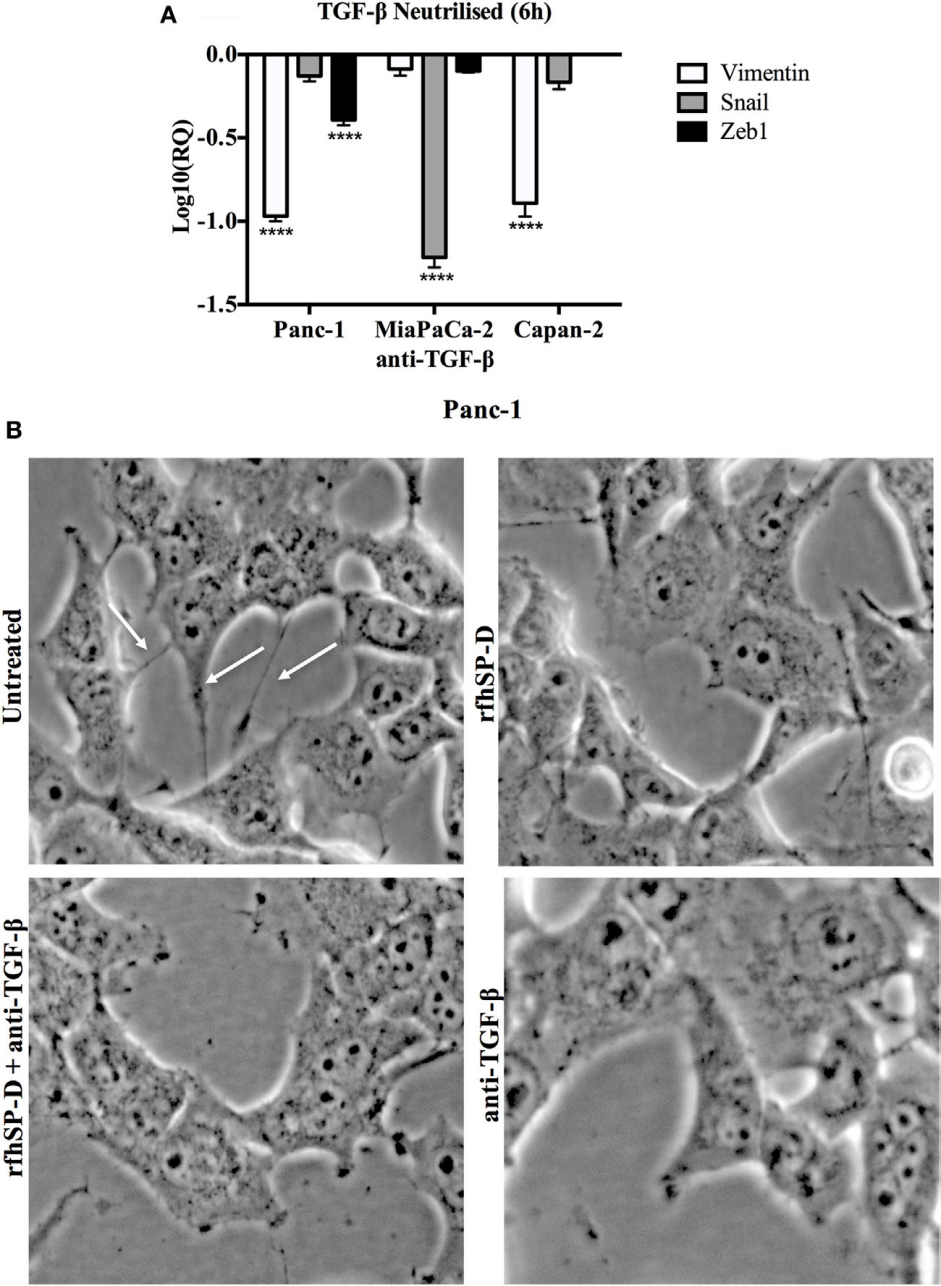
**(A)** TGF-β caused downregulation of epithelial-to-mesenchymal transition (EMT) markers. All cell lines were incubated with TGF-β neutralizing antibody for 6 h to analyze the gene expression of Vimentin, Snail, and Zeb1, which showed that all EMT markers were downregulated in a manner similar to recombinant fragment of human SP-D (rfhSP-D) treatment. Significant values were considered based on ****p < 0.0001 between treated and untreated samples. **(B)** Analysis of images taken at 6 h following the treatment with rfhSP-D, anti-TGF-β, both rfhSP-D and anti-TGF-β and untreated begins to show less branches (highlighted with white arrows in UT) and decreased cell movement in all the treated samples as compared to untreated control, which translates into significant difference by 24 h as shown in cell invasion assay.

To further establish the TGF-β association, we used neutralizing antibody in order to block TGF-β in all cell lines and assessed the expression of these EMT markers. All cells lines were incubated with TGF-β neutralizing antibody for 6 h and the mRNA expression for EMT markers (Vimentin, Snail, and Zeb1) was measured by qPCR. Interestingly, blocking TGF-β showed similar downregulation trend as seen for the rfhSP-D treatment, which further validated that rfhSP-D treatment caused downregulation of TGF-β, which in turn suppressed EMT regulators (Figure 7A). The images were taken for Panc-1 cell line to analyze the cell morphological differences following treatment with rfhSP-D, anti-TGF-β antibody, or rfhSP-D + anti-TGF-β together, to compare with those cells that were untreated at 6 h (Figure 7B). Less branches and cell movement were observed in all the treated cells as compared to the untreated control, which suggested that suppressed EMT effects become apparent as early as 6 h following the rfhSP-D treatment. Interestingly, but not surprisingly, the effect was even more pronounced when rfhSP-D (20 µg/ml) and anti-TGF-β were added together (Figure 7B).

## Discussion

SP-D was originally considered to be a lung-specific hydrophilic surfactant protein that agglutinated a diverse range of pathogens (viruses, bacteria, and fungi). It also acts as an opsonin, enhancing pathogens’ phagocytosis and subsequent killing (31). However, its ability to bind allergens (of *Aspergillus fumigatus* and house dust mite) and inhibit allergen IgE interaction and subsequent histamine release from sensitized basophils raised the possibility that SP-D could be involved in dampening pulmonary hypersensitivity (32, 33). In a murine model of allergic hypersensitivity, rfhSP-D treatment lowered specific IgE level, reduced pulmonary and peripheral eosinophilia, and caused Th2 to Th1 polarization (34). Subsequently, it was found that eosinophils derived from allergic patients, but not non-sensitized eosinophils derived from healthy subjects, underwent apoptosis when treated with rfhSP-D *in vitro* (28). An eosinophilic leukemic cell line, AML14.3D10, was thus examined to understand the mechanism of apoptosis induction by rfhSP-D (21). It revealed that levels of various apoptotic markers, such as activated p53, cleaved caspase 9, PARP, and G2/M checkpoints were considerably increased following rfhSP-D treatment, in addition to reduced levels of survival factors including HMGA1 (21, 35). Experiments with activated PBMCs also mirrored these observations (36). These studies have lent credence to the idea that SP-D is an innate immune surveillance molecule, especially outside lungs.

Hasegawa et al. have earlier shown that by interfering with EGF–EGFR interaction, SP-D can downregulate EGF signaling in A549 and H441 human lung adenocarcinoma cells (22), thus suppressing proliferation, migration, and invasiveness. Recently, Kaur et al. have shown that rfhSP-D can induce apoptosis in a range of pancreatic cancer cell lines *via* TNF-α/Fas-mediated pathway irrespective of the p53 status of the cells (29). Using Panc-1 (p53^mt^), MiaPaCa-2 (p53^mt^), and Capan-2 (p53^wt^) pancreatic cancer cell lines, we found that rfhSP-D treatment for 24 h caused growth arrest in G1 cell cycle phase and triggered upregulation of pro-apoptotic markers, TNF-α, and Fas, which eventually caused apoptosis by 48 h (29).

In the current study, we wanted to assess the effects of rfhSP-D on PDA cell lines at earlier time points of the rfhSP-D treatment with a view that EMT may potentially be modulated. We have earlier reported that the binding of rfhSP-D to PDA cells is calcium-dependent but not sugar-dependent. Thus, it is likely that CRDs are involved in binding to target ligand on the cancer cell surface *via* protein–protein interaction. As a follow up study (29), we mainly focused on investigating the effects of rfhSP-D on EMT on these cancer cells. We have earlier reported using all necessary controls (including BSA and full length SP-D) and experiments (cell binding and cell viability assays), which established the specificity of rfhSP-D. BSA did not have any effect on PDA cell lines while full-length native SP-D purified from the lung lavage of alveolar proteinosis patients, was as good as rfhSP-D (29). Due to the ease of the production of large amounts of rfhSP-D, compared to full length SP-D, we have carried out these experiments using rfhSP-D, which is well characterized in the literature as a potential therapeutic molecule.

EMT induction is characterized by morphological alterations, enhanced motility, reduced cell–cell contact (17), and upregulation of mesenchymal markers, such as Vimentin (8), Snail (18), and Zeb1 (9). In this study, we report, for the first time, a novel anti-EMT role of human SP-D, which interferes with TGF-β induced EMT by blocking Smad phosphorylation, using pancreatic cancer cell lines, Panc-1, and MiaPaCa-2, and Capan-2. The rfhSP-D treatment of these cell lines for up to 24 h prevented their morphological alterations associated with EMT, reduced tumor cell invasion in the matrigel, downregulated TGF-β production, and downregulated key mesenchymal gene expression, such as Vimentin, Zeb1, and Snail. These hierarchical observations suggested that rfhSP-D can attenuate the TGF-β pathway to suppress EMT.

Overexpression of TGF-β1 in tumor microenvironment suppresses immune surveillance and facilitates the escape, migration, and increased resistance to anti-tumor immune responses (37–39). TGF-β exerts anti-proliferative effects on natural killer cells and cytotoxic T cells (40–43). TGF-β1 has also been shown to upregulate vascular endothelial growth factor (VEGF) production, thus, enhances the liver metastasis of pancreatic cancer by regulating angiogenesis in a mouse model (44). TGF-β binds to heterotetrameric receptor complex consisting of type I (TβRI/ALK5) and type II receptors (TβRII) to activate downstream SMADs, which act as signal transducers from the receptors to the nucleus (45, 46). TGF-β expression, as revealed by qPCR and Western blot, was downregulated in Panc-1 and MiaPaCa-2 cell lines following rfhSP-D treatment. Capan-2 was unaffected, which may be attributed to its attenuated TGF-β signaling (47), thus, this cell line acted as a negative control in this study. In addition, minimal presence of SMAD 2/3 was detected in the cytoplasm and no translocation to the nucleus was detected in the rfhSP-D-treated cells as compared to untreated Panc-1 and MiaPaCa-2 cell lines. This suggested that the downregulation of TGF-β had prevented the recruitment of SMADs, the signal transduction molecules into the nucleus. Previous studies have shown that TGF-β stimulation causes the phosphorylation of SMAD 2/3, which then accumulate in nucleus to drive transcription of various target genes (46), including key mesenchymal markers, such as Vimentin (10) and Snail (10, 48) in pancreatic cancer, which regulate EMT by either direct or indirect effect on epithelial cell adhesion marker such as E-cadherin (49).

The treatment of Panc-1, MiaPaCa-2, and Capan-2 with rfhSP-D resulted in downregulation of Snail and Zeb1; however, Vimentin downregulation was only observed in Panc-1 and MiaPaCa-2 cells. Capan-2 has previously been shown not to express Vimentin (50). This suggests that due to downregulation of TGF-β, phosphorylation of SMADs is affected, since no Smad2/3 was seen either in the cytoplasm or nucleus, which then further causes the downregulation of EMT inducing genes resulting in a static state and loss of key EMT-associated morphological features. Moreover, when TGF-β was blocked for 6 h by a neutralizing antibody, gene expressions of Vimentin, Snail, and Zeb1 were downregulated similar to rfhSP-D treatment, re-affirming that rfhSP-D suppressed EMT by downregulating TGF-β pathway. These results, thus, explain the significantly reduced invasion of rfhSP-D-treated Panc-1 and MiaPaCa-2 in the matrigel matrix pre-coated with extracellular matrix proteins that promotes invasion, whereas the non-invasive Capan-2 cells remained unaffected.

In a previous study, stable and short hairpin RNA-mediated Zeb1-knockdown in Panc-1 and MiaPaCa-2 cells with overexpressed Zeb1 showed a reduced sphere formation, a hallmark of self-renewal and differentiation (9). In addition, Zeb1 knock-out in orthotopic mouse xenograft models significantly affected the tumor growth and EMT by switching the expression of Vimentin and E-cadherin (9). Similarly, downregulation of Snail in Panc-1 cells has been shown to increase its sensitivity to chemotherapeutics or radiation (51). Therefore, downregulation of mesenchymal markers can be crucial to target the EMT driven by TGF-β signaling pathway, which is a critical event in the progression of pancreatic cancer. In view of this study, rfhSP-D offers potentially a novel therapeutic approach to restore the epithelial phenotype in pancreatic cancer. Interestingly, rfhSP-D was most effective in the invasive cancer cells lines, i.e., Panc-1 and MiaPaCa-2, unlike in the non-invasive Capan-2 cell line, which indicates that it selectively targets the mesenchymal-differentiated cells. This is consistent with previous studies where rfhSP-D did not affect the eosinophils derived from healthy individual whereas it induced apoptosis in the sensitized eosinophils from allergic patients (21, 28). Targeting EMT pathway using rfhSP-D could not only lead to decreased invasiveness but also promote drug sensitivity. As mentioned earlier, Zeb1 knockout Panc-1 clones were more susceptible to chemotherapy and their proliferation was significantly reduced (9). Silencing of Zeb1 in Panc-1 and MiaPaCa-2 cells reversed the E-cadherin expression and a significantly increased apoptotic cell death was observed following gemcitabine, 5-FU, and cisplatin treatment (52). Therefore, it is important to explore the EMT suppressor role of rfhSP-D in combination with conventional chemotherapy as a therapeutic strategy against pre-malignant stages of solid tumor progression such as PDA.

## Author Contributions

AK carried out most of the crucial experiments with support from MR. SS provided key data, reagents, and ideas. UK led the project and prepared the manuscript together with AK and SS.

## Conflict of Interest Statement

The authors declare that the research was conducted in the absence of any commercial or financial relationships that could be construed as a potential conflict of interest.
